# Socio-Demographic, Social-Cognitive, Health-Related and Physical Environmental Variables Associated with Context-Specific Sitting Time in Belgian Adolescents: A One-Year Follow-Up Study

**DOI:** 10.1371/journal.pone.0167553

**Published:** 2016-12-09

**Authors:** Cedric Busschaert, Nicola D. Ridgers, Ilse De Bourdeaudhuij, Greet Cardon, Jelle Van Cauwenberg, Katrien De Cocker

**Affiliations:** 1 Department Movement & Sport Sciences, Ghent University, Ghent, Belgium; 2 Research Foundation Flanders (FWO), Brussels, Belgium; 3 Centre for Physical Activity and Nutrition Research, Deakin University, Burwood Melbourne, Australia; 4 Department Public Health, Ghent University, Ghent, Belgium; 5 Department of Human Biometry and Biomechanics, Vrije Universiteit Brussel, Brussels, Belgium; Vanderbilt University, UNITED STATES

## Abstract

**Introduction:**

More knowledge is warranted about multilevel ecological variables associated with context-specific sitting time among adolescents. The present study explored cross-sectional and longitudinal associations of ecological domains of sedentary behaviour, including socio-demographic, social-cognitive, health-related and physical-environmental variables with sitting during TV viewing, computer use, electronic gaming and motorized transport among adolescents.

**Methods:**

For this longitudinal study, a sample of Belgian adolescents completed questionnaires at school on context-specific sitting time and associated ecological variables. At baseline, complete data were gathered from 513 adolescents (15.0±1.7 years). At one-year follow-up, complete data of 340 participants were available (retention rate: 66.3%). Multilevel linear regression analyses were conducted to explore cross-sectional correlates (baseline variables) and longitudinal predictors (change scores variables) of context-specific sitting time.

**Results:**

Social-cognitive correlates/predictors were most frequently associated with context-specific sitting time. Longitudinal analyses revealed that increases over time in considering it pleasant to watch TV (p < .001), in perceiving TV watching as a way to relax (p < .05), in TV time of parents/care givers (p < .01) and in TV time of siblings (p < .001) were associated with more sitting during TV viewing at follow-up. Increases over time in considering it pleasant to use a computer in leisure time (p < .01) and in the computer time of siblings (p < .001) were associated with more sitting during computer use at follow-up. None of the changes in potential predictors were significantly related to changes in sitting during motorized transport or during electronic gaming.

**Conclusions:**

Future intervention studies aiming to decrease TV viewing and computer use should acknowledge the importance of the behaviour of siblings and the pleasure adolescents experience during these screen-related behaviours. In addition, more time parents or care givers spent sitting may lead to more sitting during TV viewing of the adolescents, so that a family-based approach may be preferable for interventions. Experimental study designs are warranted to confirm the present findings.

## Introduction

Sedentary behaviour (SB), defined as any waking activity characterized by an energy expenditure ≤ 1.5 metabolic equivalents (METs) performed in a sitting or reclining posture [1], has received increasing attention in terms of chronic disease prevention [2]. Too much sitting, independently of physical activity, has been associated with obesity, higher blood pressure and total cholesterol, lower levels of physical fitness, lower bone mineral acquisition, lowered scores for self-esteem and prosocial behaviour and decreased academic achievement in adolescents [3, 4]. Importantly, decreasing total sitting time in adolescence may be important, as there is increasing evidence that high levels of sitting time, especially TV viewing, during adolescence are associated with negative health outcomes in young/mid-adulthood [5–7]. Besides decreasing total sitting time, interrupting long periods of sitting may also have positive health effects among youth [8–11]. Despite the rising evidence of these negative health effects, adolescents have high levels of objectively-measured (activPAL-derived) total daily sitting time (10 h.d^−1^) [12]. Sitting time during TV viewing, computer use, playing video games and motorized transport account for a large proportion of adolescents’ leisure time sitting, and have increased over the past 20 years [13–15].

As sitting is a complex behaviour that occurs in multiple contexts during leisure time (e.g. TV viewing, computer use and motorized transport) [16], gathering context-specific information is also warranted. For example, it is assumed that screen-related sitting time has a stronger association with adiposity compared to motorized transport or sitting during hobbies, as an unhealthy eating behaviour may be an important contributor in the association between screen-related sitting time and adiposity [17]. Esteban-Cornejo et al. [18] found that adolescents had high levels of sitting time during screen-related behaviours in leisure time of which on average 89 minutes/day, 33 minutes/day and 46 minutes/day were spent during TV viewing, playing computer/video games and surfing on the internet, respectively. Further, adolescents spend on average 42 minutes/day sitting during motorized transport, typically travelling to and from school [19]. In order to inform interventions to decrease adolescents’ sitting time during common leisure behaviours (i.e. TV viewing, computer use, electronic gaming and motorized transport), it is important to identify modifiable variables that are associated with sitting time.

Previous research highlighted that correlates (cross-sectional) and predictors (longitudinal) differed between particular contexts of sitting time in adolescence and early-adulthood [20, 21]. Therefore, it is recommended to identify variables associated with context-specific sitting time across multiple levels based on a socio-ecological model [22]. Recently, a systematic review revealed that predictors at the individual level (e.g. age, socio-economic status, maturation) were more consistently associated with total sitting time and screen time compared with variables at the interpersonal, environmental and policy level [23]. Socio-demographic variables are useful in identifying high levels of (context-specific) sitting time in population subgroups such as men versus women, however, more research is warranted to identify modifiable variables that will guide efforts to intervene. Therefore, more research is warranted to map a broad list of associated multilevel ecological variables of context-specific sitting time [23]. To date, few studies examined whether cross-sectional correlates differed from longitudinal predictors which may reveal information on the usability of cross-sectional correlates in future intervention studies aiming to change sitting time [24]. Therefore, research that identifies both cross-sectional and longitudinal associations with context-specific sitting time will be valuable to identify if cross-sectional correlates actually cause change in context-specific sitting time.

The objective of the present longitudinal study with a follow-up period of one year was to investigate multilevel ecological correlates and predictors of context-specific sitting time among Belgian adolescents. First, cross-sectional correlates (i.e. socio-demographic, social-cognitive, physical environmental and health-related variables at baseline) of adolescents’ context-specific sitting time at baseline (i.e. TV-viewing, computer use, electronic gaming and motorized transport) were identified (aim A). Secondly, it was examined whether changes in these socio-demographic, social-cognitive, physical environmental and health-related variables from baseline to follow-up predicted changes in adolescents’ context-specific sitting (aim B).

## Materials and Methods

### Study design

In the present longitudinal study data were gathered using a follow-up period of one year. Baseline data were gathered between February 2014 and May 2014, and follow-up data were collected between March 2015 and June 2015.

### Subjects and procedures

Adolescents were recruited through secondary schools located in Flanders, Belgium. Sixteen schools were contacted via mail and/or telephone. All headmasters/principals received an information letter with a short explanation (background, objectives and practical information) about the study. Seven schools agreed to participate (response rate: 44%), whereupon a meeting was planned with every headmaster/principal separately. During this meeting, the headmaster selected the participating classes (consisting of classes of each type of education provided at the school (i.e. general secondary education, technical secondary education and/or vocational secondary education) and of different age groups (n = 566 adolescents eligible to participate in the selected classes). Belgium secondary schools mostly offer these types of education so students can choose between a general education as preparation for high school or university, technical education with more focus on technical and practical aspects or vocational education to learn specific skills for a certain job [25]. Additionally, dates when questionnaires could be delivered were agreed upon. It was important that no students in their final year were selected to participate in the longitudinal study so that students could be contacted through the same school to participate in the follow-up measurement. Adolescents who agreed to participate in the present study completed a questionnaire during class time at school. To be included in the study, participants had to be aged 12–18 years at baseline and Dutch-speaking. Fifty-three adolescents were not included in the analyses, due to several reasons: not present at the moment when filling out the questionnaire (n = 29), no permission provided from parents/care givers (n = 18) or incomplete data (n = 6). This resulted in a final sample of 513 adolescents at baseline (participation rate: 90.64%).

At follow-up, the headmasters of the participating schools were contacted in order to distribute the questionnaire to the adolescents who participated at baseline. As adolescents of the particular classes at baseline were located in different classes at follow-up, the headmasters decided that only classes with the largest group of adolescents of each particular class at baseline should be invited to participate at the follow-up measurement, resulting in a minimal load for the schools, as otherwise many classes with few participating students had to be included. Consequently, from the 513 participants at baseline, 378 adolescents remained eligible to participate at follow-up (135 adolescents changed class at follow-up or left the participating school). Furthermore, 38 adolescents were not included in the analyses due to several reasons (i.e. not present at the moment when filling out the questionnaire: n = 30; refused to participate: n = 2; incomplete data: n = 6). This resulted in a final sample of 340 adolescents participating both at baseline and follow-up (retention rate: 66.28%).

Adolescents’ parents/care givers were asked to consent prior to the start of the study. At baseline and follow-up, a passive written consent procedure was used, so parents/care givers who did not allow their child to participate returned a signed letter stating they refused participation of their child. The other parents/care givers provided consent by not returning the letter. Furthermore, at both measurement stages, adolescents themselves could refuse to fill out the questionnaires and this without any consequences (voluntary participation), but none of the adolescents refused. The study protocol was approved by the Ghent University Hospital Ethics Committee.

### Measures

Adolescents completed a questionnaire about context-specific sitting time and its potential socio-demographic, social-cognitive, health-related and physical environmental associated variables. The questionnaire has shown acceptable validity and moderate test-retest reliability for total sitting time on an average day [12]. Test-retest reliability for the included context-specific sitting time outcomes (i.e. TV viewing, computer use, electronic gaming and motorized transport) showed lower results for sitting during electronic gaming and motorized transport [12]. The associated variables (correlates/predictors) of context-specific sitting time which were shown to have moderate-to-excellent reliability were included in the analyses of the present study [12]. Therefore, some social-cognitive variables were excluded from the analyses as they had poor reliability (see Table 1).

**Table 1:** Overview of the included item-specific social-cognitive variables.

	Item questionnaire	Baseline (mean ± SD)	Follow-up (mean ± SD)	Change score follow-up—baseline (mean ± SD)
**TV viewing**				
Attitude 1^(a)^	I think watching TV is pleasant	4.16 ± 0.84	3.97 ± 0.93	-0.19 ± 1.02
Attitude 2^(a)^	Watching TV takes time away from doing other important things	2.85 ± 1.09	2.76 ± 1.13	-0.07 ± 1.25
Attitude 3^(a)^	I enjoy watching TV for many hours at a time	2.64 ± 1.16	2.76 ± 1.12	0.10 ± 1.27
Attitude 4^(a)^	Watching TV is my way to relax	3.31 ± 1.15	3.22 ± 1.03	-0.09 ± 1.20
Self-efficacy 1^(a)^	I consider it possible to reduce my TV time	2.82 ± 1.16	2.71 ± 1.17	-0.13 ± 1.38
Self-efficacy 2^(a)^	I consider it possible to turn off the TV during weekend days until 5:00 p.m.	3.20 ± 1.44	3.22 ± 1.37	0.00 ± 1.48
Self-efficacy 3^(a)^	I consider it possible to turn off the TV during meals	3.89 ± 1.40	3.85 ± 1.32	-0.04 ± 1.22
Norm^(a)^	I think that I spend too much time watching TV	2.05 ± 1.03	1.96 ± 1.04	-0.11 ± 1.15
Social norm^(a)^	My family members think I spend too much time watching TV	2.11 ± 1.10	1.95 ± 1.06	-0.15 ± 1.12
Social support 1^(a)^	My family members encourage me to watch less TV	2.04 ± 1.06	2.06 ± 1.06	0.00 ± 1.15
Social support 2^(a)^	My friends encourage me to watch less TV	1.46 ± 0.80	1.61 ± 0.93	0.15 ± 1.08
Modelling 1^(b)^	How long, on average, do your parents/care givers spend watching TV in leisure time?	131.45 ± 86.59	128.00 ± 86.90	-3.07 ± 76.79
Modelling 2^(b)^	How long, on average, do your siblings spend watching TV in leisure time?	150.59 ± 96.28	140.83 ± 89.89	-6.35 ± 94.75
Parental rules^(c)^	Do your parents/care givers have rules about how many hours per day you are allowed to watch TV?	0.17 ± 0.38	0.12 ± 0.32	-0.07 ± 0.36
**PC-use**				
Attitude 1^(a)^	I think using a computer is pleasant in leisure time	3.86 ± 1.04	3.67 ± 1.07	-0.18 ± 1.12
Attitude 2^(a)^	Using a computer takes time away from doing other important things	2.72 ± 1.16	2.66 ± 1.11	-0.06 ± 1.35
Attitude 3^(a)^	I enjoy using a computer for many hours at a time	3.08 ± 1.28	3.03 ± 1.22	-0.06 ± 1.29
Attitude 4^(a)^	Using a computer is my way to relax	3.23 ± 1.26	3.09 ± 1.22	-0.15 ± 1.29
Self-efficacy 1^(a)^	I consider it possible that I do not use a computer for some days in the week (leisure time)	3.03 ± 1.37	2.98 ± 1.33	-0.08 ± 1.56
Self-efficacy 2^(a)^	I consider it possible to reduce my computer time in leisure time	3.02 ± 1.20	2.84 ± 1.20	-0.19 ± 1.45
Norm^(a)^	I think that I spend too much time using a computer in leisure time	2.10 ± 1.11	2.18 ± 1.06	0.08 ± 1.22
Social norm^(a)^	My family members think I spend too much time using a computer^(*)^	/	/	/
Social support 1^(a)^	My family members encourage me to spend less time using a computer in leisure time	2.17 ± 1.16	2.16 ± 1.13	-0.03 ± 1.24
Social support 2^(a)^	My friends encourage me to spend less time using a computer in leisure time	1.61 ± 0.87	1.76 ± 0.94	0.13 ± 1.11
Modelling 1^(b)^	How long, on average, do your parents/care givers sit/lying down when using the computer in leisure time?	97.45 ± 100.97	94.08 ± 96.09	-3.04 ± 96.59
Modelling 2^(b)^	How long, on average, do your siblings sit when using the computer in leisure time?	124.51 ± 106.89	123.17 ± 110.67	6.33 ± 122.13
Parental rules^(c)^	Do your parents/care givers have rules about how many hours per day you are allowed to use a computer?	0.22 ± 0.42	0.13 ± 0.33	-0.09 ± 0.39
**Electronic gaming**				
Attitude 1^(a)^	I think playing computer/video games is pleasant	3.92 ± 1.27	3.86 ± 1.28	-0.12 ± 1.01
Attitude 2^(a)^	I enjoy playing computer/video games for many hours at a time	2.99 ± 1.48	3.07 ± 1.42	0.03 ± 1.18
Attitude 3^(a)^	Playing computer/video games takes time away from doing other important things	2.72 ± 1.29	2.70 ± 1.29	-0.03 ± 1.55
Attitude 4^(a)^	Playing computer/video games is my way to relax	3.21 ± 1.38	3.17 ± 1.42	-0.09 ± 1.27
Self-efficacy 1^(a)^	I consider it possible to reduce my time playing computer/video games^(*)^	/	/	/
Norm^(a)^	I think that I spend too much time playing computer/video games	2.10 ± 1.16	2.09 ± 1.10	0.00 ± 1.19
Social norm^(a)^	My family members think I spend too much time playing computer/video games	2.49 ± 1.37	2.34 ± 1.29	-0.19 ± 1.22
Social support 1^(a)^	My family members encourage me to spend less time playing computer/video games	2.29 ± 1.29	2.26 ± 1.21	0.02 ± 1.30
Social support 2^(a)^	My friends encourage me to spend less time playing computer/video games	1.62 ± 0.96	1.69 ± 0.94	0.05 ± 1.12
Modelling 1^(b)^	How long, on average, do your parents/care givers sit when playing computer/video games in leisure time?	16.18 ± 41.62	23.88 ± 61.86	3.75 ± 63.34
Modelling 2^(b)^	How long, on average, do your siblings sit when playing computer/video games in leisure time?	101.16 ± 107.44	96.43 ± 114.52	-7.83 ± 103.43
Parental rules^(c)^	Do your parents/care givers have rules about how many hours per day you are allowed to play computer/video games?	0.26 ± 0.44	0.18 ± 0.39	-0.09 ± 0.37
**Motorized transport**				
Attitude 1^(a)^	I think using motorized transport is pleasant	3.26 ± 1.17	3.27 ± 1.11	0.01 ± 1.29
Attitude 2^(a)^	I think it is pleasant to work (e.g. school-related work, call someone, …) or to rest as a passenger during motorized transport^(*)^	/	/	/
Attitude 3^(a)^	I feel lazy arriving at my destination after motorized transport ^(^)^	3.11 ± 1.29	3.25 ± 1.20	0.14 ± 1.57
Self-efficacy 1^(a)^	I consider it possible to get off the bus/metro spontaneously 1 stop before my destination and to walk the remaining distance	2.45 ± 1.33	2.40 ± 1.30	-0.06 ± 1.52
Self-efficacy 2^(a)^	I consider it possible to take the bicycle or to go by foot spontaneously even if it is possible to use a bus/metro or ride in a car^(*)^	/	/	/
Norm^(a)^	I think that I spend too much time using motorized transport	2.08 ± 1.06	2.12 ± 1.02	0.02 ± 1.22
Social norm^(a)^	My family members think I spend too much time using motorized transport	1.69 ± 0.91	1.85 ± 1.00	0.15 ± 1.20
Social support 1^(a)^	My family members encourage me to use (more often) active transport (to bicycle or to walk)	2.51 ± 1.36	2.50 ± 1.26	-0.05 ± 1.49
Social support 2^(a)^	My friends encourage me to use (more often) active transport (to bicycle or to walk)	1.86 ± 1.08	2.05 ± 1.12	0.17 ± 1.28
Modelling 1^(d)^	The most chosen transportation possibility to go to work/school from my parents/care givers is …	0.85 ± 0.35	0.84 ± 0.36	-0.01 ± 0.29
Modelling 2^(d)^	The most chosen transportation possibility in leisure time from my parents/care givers is …	0.77 ± 0.42	0.80 ± 0.40	0.04 ± 0.44
Modelling 3^(d)^	The most chosen transportation possibility to go to work/school from my siblings is …	0.55 ± 0.50	0.58 ± 0.50	0.00 ± 0.43
Modelling 4^(d)^	The most chosen transportation possibility in leisure time from my siblings is …	0.58 ± 0.49	0.67 ± 0.47	0.07 ± 0.56

Note: (*) indicates an item that is not included due to low test-retest reliability. (^) indicates an item that was recoded because of negative scoring. Abbreviations: PC-use (computer use).

*Answering categories*^*(a)*^*: strongly disagree; somewhat disagree; neutral; somewhat agree; strongly agree [1–5]*

*Answering categories*^*(b)*^*: 0 min/day; 7.5 min/day; 22.5 min/day; 45 min/day; 90 min/day; 150 min/day; 210 min/day; 270 min/day; 330 min/day; 390 min/day; 450 min/day*

*Answering categories*^*(c)*^*: no; yes [0;1]*

*Answering categories*^*(d)*^*: active transport (walking, bicycling); motorized transport [0;1]*

### Sitting time variables (outcome)

**Context-specific sitting** (i.e. TV-viewing, computer use, electronic gaming and motorized transport) was measured identically at baseline and follow-up. Participants reported their average daily sitting time during the past seven days during TV viewing, computer use (laptop, desktop, tablet and internet use on smartphone) and electronic gaming in leisure time (separately for weekday and weekend day), motorized transport in leisure time (separately for weekday and weekend day) and motorized transport to and from school. The following answer categories were used separately for TV viewing, computer use and electronic gaming: ‘none’, ‘1–15 minutes/day’, ‘15–30 minutes/day’, ‘30–60 minutes/day’, ‘1–2 hours/day’, ‘2–3 hours/day’, ‘3–4 hours/day’, ‘4–5 hours/day’, ‘5–6 hours/day’, ‘6–7 hours/day’ or ‘more than 7 hours/day’. The answer categories for motorized transport in leisure time and motorized transport to and from school were slightly different (‘none’, ‘1–15 minutes/day’, ‘15–30 minutes/day’, ‘30–45 minutes/day’, ‘45–60 minutes/day’, ‘60–90 minutes/day’, ‘90–120 minutes/day’, ‘2–2.5 hours/day’, ‘2.5–3 hours/day’, ‘3–4 hours/day’, ‘4–5 hours/day’, ‘5–6 hours/day’, ‘6–7 hours/day’ or ‘more than 7 hours/day’).

### Potential associated variables of context-specific sitting time

The potential associated variables, which were measured identically at baseline and follow-up, were divided into four levels: socio-demographic, social-cognitive, physical environmental and health-related variables.

#### Socio-demographic variables

The socio-demographic variables examined were: *family situation*, *parental education*, *sex*, *residential area*, *having siblings*, *age* (determined based on date of birth and date of filling out questionnaire) and *type of education* (detailed information provided in Table 2).

**Table 2:** Overview of the included (changes in) socio-demographic variables.

	Questionnaire item	Original answer category	Recoded variables for cross-sectional analyses (baseline)	Longitudinal analyses	
				Recoding (% or mean ± SD)	New variables based on recoding
Family situation	Where do you live?	1 = both parents	0 [other = 28.5%]	-	-
		2 = half the time with your mother and other half with your father			
		3 = exclusively with your mother			
		4 = exclusively with your father			
		5 = mother and her new partner	1 [both parents = 71.5%]		
		6 = father and his new partner			
		7 = grandparents or other family member			
		8 = institution or boarding school			
		9 = other			
Parental education	What is the highest achieved diploma or studies doing at the moment from your father and mother (or cohabiting partner)?	1 = primary school	0 [no higher education of both parents = 45.5%]	-	**-**
		2 = secondary education			
		3 = higher education, non-university	1 [at least one parent in possession of diploma of higher education = 54.5%]		
		4 = higher education, university			
Sex	What is your sex?	1 = male	0 [female = 35.7%];	-	**-**
		2 = female	1 [male = 64.3%]		
Residential area	In which type of area do you live?	1 = countryside	0 [countryside and village/town = 80.7%]	0→0 or 1→1 (stable = 87.6%)	0 = stable
		2 = village or town			
		3 = cities suburbs	1 [cities suburbs and city = 19.3%]	1→0 (decrease = 6.7%)	1 = decrease
		4 = city		0→1 (increase = 5.8%)	2 = increase
Having siblings	How many siblings do you have?	… brothers and/or … sisters	0 [no siblings = 12.1%]	-	**-**
			1[having siblings = 87.9%]		
Age	Date of birth and date of filling out questionnaire	day/month/year	… years old	-	**-**
Type of education	In which type of education do you take classes?	1 = arts education	0 [others, i.e. vocational or technical secondary education = 66.7%]	0→0 or 1→1 (stable = 94.7%)	0 = stable
		2 = vocational secondary education			1 = change to general secondary education
		3 = technical secondary education	1[general secondary education = 33.3%]	0→1 (change to general secondary education = 0.3%	2 = change to vocational or technical secondary education
		4 = general secondary education		1→0 (change to vocational or technical secondary education = 5.0%)	

Note: “-” indicates variables that were not included in longitudinal analyses.

#### Social-cognitive variables

Social-cognitive variables (i.e. *attitude*, *self-efficacy*, *norm*, *social norm*, *social support* and *modelling*) were assessed specifically for each included context-specific sitting time. These variables were inserted individually on item level (e.g. attitude 2 TV viewing: ‘watching TV takes time away from doing other important things’; attitude 3 TV viewing: ‘I enjoy watching TV for many hours at a time’), as this ensured more specific information (see Table 1) [26]. Attitude, self-efficacy, (social) norms and social support were determined by asking participants to rate different statements with answer categories ranging from ‘strongly disagree’ (1) to ‘strongly agree’ (5). The answer categories about modelling (separately for parents/care givers and siblings) for TV viewing, computer use and electronic gaming ranged from ‘none’ (no TV viewing, computer use or electronic gaming) (1) to ‘more than 7 hours/day’ (11) (11-point Likert scale). For motorized transport, the answer categories about modelling (to work or school and in leisure time, separately for parents/caregivers and siblings) were dichotomous (i.e. active transport or motorized transport). Further, *parental rules* concerning time adolescents spent sitting during TV viewing, computer use and electronic gaming were included (yes/no question).

#### Physical environmental variables

Physical environmental variables were assessed specifically for each context of sitting time (see Table 3). Adolescents indicated the number of devices for TV viewing, computer use and electronic gaming available in the household that they operated themselves with answer categories ranging from ‘none’ (1) to ‘more than 5’ (7) (7-point Likert scale). For TV-viewing, the variable ‘*TV set*’ referred to the number of televisions and the variable ‘*other TV-viewing equipment*’ was calculated by summing the number of laptops, desktop computers, smartphones and tablets. For computer use, the number of laptops and desktop computers were summed to calculate the variable ‘*computer equipment*’. The variable ‘*other equipment for computer use*’ was calculated by summing the number of smartphones and tablets. For electronic gaming, the variable ‘*equipment for playing games’* was obtained by summing the number of (portable) electronic gaming consoles and the variable ‘*other equipment for playing games’* was calculated by summing the number of smartphones, tablets, laptops and desktop computers. Three additional physical environmental variables were obtained regarding TV viewing. Adolescents had to indicate if they agreed (‘strongly disagree’ (1) to ‘strongly agree’ (5)) with the following items on a five-point Likert scale: ‘*the remote controller (TV) can always be found closely to me when I need it*’ and ‘*the couches at our place are comfortable to sit for a long time*’. Also, the variable *‘TV in bedroom’* was included in the analyses regarding TV viewing and indicated whether the adolescents had a TV in their bedroom (dichotomous variable). For motorized transport, adolescents reported the *number of all operational motorized vehicles* available in the household (e.g. cars or motorbikes).

**Table 3:** Overview of the included physical environmental variables.

	Items	Baseline (mean ± SD)	Follow-up (mean ± SD)	Change-score follow-up—baseline (mean ± SD)
TV set	How many TV’s do you use and are present at your home?^(1)^	2.33 ± 1.14	2.32 ± 1.15	-0.01 ± 0.80
Other TV-viewing equipment	How many of the following electronic devices do you use and are present at your home?	5.90 ± 3.24	6.12 ± 3.21	0.20 ± 3.34
	a) laptops^(1)^			
	b) desktop computers^(1)^			
	c) smartphones^(1)^			
	d) tablets^(1)^			
TV in bedroom	TV in bedroom?^(2)^	0.45 ± 0.50	0.46 ± 0.50	0.00 ± 0.36
Remote controller	The remote controller (TV) can always be found closely to me when I need it^(3)^	3.82 ± 1.25	3.84 ± 1.16	-0.00 ± 1.38
Sitting furniture (couches)	The couches at our place are comfortable to sit for a long time^(3)^	4.39 ± 0.96	4.25 ± 0.93	-0.12 ± 1.10
PC equipment	How many of the following electronic devices do you use and are present at your home?	3.00 ± 1.63	2.85 ± 1.54	-0.16 ± 1.71
	a) laptops^(1)^			
	b) desktop computers^(1)^			
Other equipment for computer use	How many of the following electronic devices do you use and are present at your home?	2.91 ± 2.13	3.30 ± 2.29	0.41 ± 2.31
	a) smartphones^(1)^			
	b) tablets^(1)^			
Equipment for playing games	How many of the following electronic devices do you use and are present at your home?	3.01 ± 2.26	2.73 ± 2.17	-0.27 ± 2.00
	a) non-portable gaming consoles^(1)^			
	b) portable gaming consoles^(1)^			
Other equipment for playing games	How many of the following electronic devices do you use and are present at your home?	5.90 ± 3.24	6.12 ± 3.21	0.20 ± 3.34
	a) smartphones^(1)^			
	b) tablets^(1)^			
	c) laptops^(1)^			
	d) desktop computers^(1)^			
Motorized vehicles	How many operational motorized vehicles are there present in the household, even the ones you do not use yourself?^(4)^	2.60 ± 1.74	2.66 ± 1.74	0.04 ± 1.47

Note: PC (computer), TV (television).

*Answering categories*^*(1)*^*: ‘none’, ‘1’, ‘2’, ‘3’, ‘4’, ‘5’ or ‘more than 5’ [0–6]*

*Answering categories*^*(2)*^*: ‘no’, ‘yes’ [0–1]*

*Answering categories*^*(3)*^*: ‘strongly disagree’, ‘somewhat disagree’, ‘neutral’, ‘somewhat agree’, ‘strongly agree’ [1–5]*

*Answering categories*^*(4)*^*: open-ended question*

#### Health-related variables

Health-related variables were documented using subjective measurements (see Table 4). Adolescents rated their *general health* on a five-point Likert scale (‘poor’ [1] to ‘excellent’ [5]) and the Patient Health Questionnaire (2-items) was used to detect *depressive symptoms* [27, 28]. *BMI* was obtained by weight/height^2^ self-reported by the adolescents.

**Table 4:** Overview of the included health-related variables.

	**Items**	**Baseline (mean ± SD)**	**Follow-up (mean ± SD)**	**Change-score follow-up—baseline (mean ± SD)**	**Recoded variable for cross-sectional analyses (baseline)**	**Recoded variable for longitudinal analyses**
General health	In general, how would you rate your health?^(1)^	3.62 ± 0.90	3.55 ± 0.93	-0.07 ± 0.91	/	/
Depressive symptoms	Over the past 2 weeks, how often have you been bothered by any of the following problems?			/	**Dummy**^**(3)**^:	0 = stable
					0 (0–2) = 89.7%	1 = developing depressive symptoms
	a) little interest or pleasure in doing things^(2)^	0.49 ± 0.73	0.62 ± 0.79			
	b) feeling down, depressed or hopeless^(2)^	0.41 ± 0.74	0.49 ± 0.82			
					1 (3–6) = 10.3%	2 = disappearance of depressive symptoms
BMI (kg/m^2^)	Self-reported height (cm) and weight (kg)	19.82 ± 2.96	20.34 ± 2.91	0.55 ± 1.66	/	/

Note: Abbreviations: BMI (Body Mass Index). *‘/’*: *not applicable*.

*Answering categories*^*(1)*^*: ‘poor’, ‘fair’, ‘good’, ‘very good’, ‘excellent’ [1–5]*

*Answering categories*^*(2)*^*: ‘not at all’, ‘several days’, ‘more than half the days’, ‘nearly every day’ [0–3]*

*The dummy variable*^*(3)*^ *is based on the sum of the sub questions ‘a’ and ‘b’*

### Potential covariate of context-specific sitting time

Total physical activity (minutes active transport to and from school, physical education at school, sports at school, sports and active transport during leisure time per day) was assessed at baseline and follow-up using the validated Flemish Physical Activity Questionnaire (FPAQ; paper and pencil form) [29].

### Data reduction

#### Sitting time variables

The midpoint values of the above-mentioned answer categories for **sitting time** were determined to obtain numerical data (e.g. 45 minutes/day regarding the answer category ‘30–60 minutes/day’). Self-reported context-specific sitting time was calculated from TV viewing during leisure time, computer use during leisure time, electronic gaming during leisure time and motorized transport to and from school and during leisure time, using the following formula: ((sitting time on a weekday * 5) + (sitting time on a weekend day * 2))/7. Total time spent in motorized transport was calculated by summing leisure time and motorized transport to and from school. The baseline measurements of context-specific sitting time were used in the cross-sectional analyses. For the longitudinal analyses, a change score for the context-specific sitting times was calculated by subtracting baseline measurements from follow-up measurements (i.e. follow-up minus baseline).

#### Potential associated variables of context-specific sitting time

Potential correlates of context-specific sitting time (i.e. cross-sectional analyses) were determined by using the baseline measurements. Furthermore, changes in socio-demographic, social-cognitive, physical environmental and health-related variables from baseline to follow-up were included as potential predictors of changes in context-specific sitting time (i.e. longitudinal analyses). BMI z-scores were used in the analyses. These scores were calculated based on Flemish reference data [30] using the LMS method [31, 32]. BMI z-scores provide a relative measure of adiposity adjusted for age and sex. It is the number of standard deviation units that a person's BMI is deviated from a mean or reference value. Change scores of potential predictors were calculated by subtracting baseline measurements from follow-up measurements (i.e. follow-up minus baseline).

The included **socio-demographic variables** at the baseline measurements were: family situation, parental education, sex, residential area, having siblings, age and type of education. For the longitudinal analyses, change scores were only calculated for residential area and type of education, due to limited variance during one year of follow-up (e.g. having siblings, family situation) or asking for a change was irrelevant (e.g. sex) in the other above-mentioned variables. Table 2 gives an overview of the socio-demographic variables (including scoring methods and descriptive statistics), which were included in the baseline measurements and as change scores.

Further, to facilitate the interpretation of the results, all **social-cognitive**, **health-related** and **physical environmental variables** were scored/recoded in the same direction (highest score is the most positive answer). A change score for each of these variables was then calculated. For the **social-cognitive variables**, item-specific values were used to determine change scores. Detailed information about the scoring properties and descriptive statistics of the social-cognitive variables are shown in Table 1. For the **health-related variables**, the change in depressive symptoms was determined on the development or disappearance of depressive symptoms from baseline to follow-up (i.e. ‘stable’, ‘developing depressive symptoms’ or ‘disappearance of depressive symptoms’) (Table 4).

#### Potential covariate of context-specific sitting time

Total physical activity was calculated by summing minutes per day spent in active transport to and from school, physical education at school, sports at school, and sports and active transport during leisure time. Total physical activity at baseline was included as a covariate in the cross-sectional analyses. For the longitudinal analyses, a change score of total physical activity (i.e. follow-up minus baseline) was used as a covariate.

### Statistical analyses

All analyses were conducted using R Studio Version 0.98.507. Multilevel modelling (three-level: adolescent; class; secondary school) was conducted in order to account for clustering of adolescents in classes in secondary schools, which is the most appropriate technique to use for hierarchical data [33]. Normality was analyzed by assessing the Q-Q plots and skewness/kurtosis [34]. Statistical significance was determined at α = 0.05.

#### Potential cross-sectional correlates of context-specific sitting time at baseline (aim A)

For the cross-sectional analyses, the statistical model used to examine the correlates of TV viewing, computer use and motorized transport was different than the model used for electronic gaming due to different distributions of these dependent variables. Generalized linear regression analyses were performed for TV viewing, computer use and motorized transport. Since these dependent variables were non-normally distributed (i.e. they were positively skewed, p<0.001) a generalized linear model with Gamma variance and log link function was used. This model yielded the best fit based on Akaike’s Information Criterion (AIC). TV viewing, computer use and motorized transport included few zero counts, however, as models with a Gamma variance and log link function do not allow zero counts, these zero counts were adjusted (+0.001). On the other hand, hurdle models were used to examine the correlates of electronic gaming, as this dependent variable was positively skewed and included many zero counts. The hurdle model consists of two separate analyses. First, a logistic regression with binomial variance and logit link function was fitted in which the relationship with the probability of a person who did sit during electronic gaming is estimated. Secondly, hurdle models examine the relationship between potential correlates and the volume of electronic gaming among those who did sit during electronic gaming. These analyses also used generalized linear models with Gamma variance and log link function, as this model yielded the best fit based on AIC.

For the analyses, a four-step procedure was used in order to identify correlates of context-specific sitting time (i.e. separately for TV viewing, computer use, electronic gaming and motorized transport). In the first step, correlation analyses were performed to test for multicollinearity between the quantitative correlates per level (i.e. socio-demographic, social-cognitive, health-related and physical environmental variables). When two correlates belonging to the same level revealed Pearson correlation coefficients >0.60 with each other [35], the correlate that showed the lowest correlation coefficient with the dependent variable (i.e. context-specific sitting time outcome) was excluded from the analyses. Following this procedure, the following correlates were removed: ‘my family members think I spend too much time watching TV’ (social norm TV viewing), ‘I think using a computer is pleasant in leisure time’ (attitude 1 computer use), ‘using a computer is my way to relax’ (attitude 4 computer use), ‘I consider it possible to reduce my computer time in leisure time’ (self-efficacy 2 computer use), ‘I think playing computer/video games is pleasant’ (attitude 1 electronic gaming), ‘playing computer/video games is my way to relax’ (attitude 4 electronic gaming) and ‘my family members encourage me to spend less time playing computer/video games’ (social support 1 electronic gaming). Secondly, four regression models for each level separately were fitted that contained all correlates within that level. In this step, only correlates that revealed p<0.10 with the context-specific sitting time outcome were retained for the next step in the analyses [36, 21]. In step 3, multicollinearity between the remaining quantitative correlates from the previous step was tested. None of these correlates showed Pearson correlation coefficients >0.60 with each other. In the final step, all correlates showing p<0.10 in the second step were pooled into one model for each context-specific sitting time. The results of the final step are presented in the results section. All cross-sectional analyses (step 1–4) were adjusted for total physical activity at baseline.

#### Changes in potential predictors related to changes in context-specific sitting time (aim B)

For the longitudinal analyses, the change scores for TV viewing, computer use, electronic gaming and motorized transport were normally distributed and, hence, general linear models were used (Gaussian variance function). The longitudinal analyses examined if the change scores of potential predictors predicted changes in context-specific sitting time.

For the longitudinal analyses, the same four-step procedure was used as described above for aim A. In step 1, multicollinearity was tested between the quantitative change scores of potential predictors per level and ‘I enjoy using a computer for many hours at a time’ (attitude 3 computer use) and ‘I consider it possible that I do not use a computer for some days in the week during leisure time’ (self-efficacy 1 computer use) were removed. Secondly, four regression models for each level separately were performed that contained all change scores of potential predictors within that level. Thirdly, no variables were removed due to multicollinearity. In step 4, the change scores of potential predictors showing p<0.10 in the second step were pooled into one model per context-specific sitting time. The results of the final step are presented in the results section. The longitudinal analyses (steps 1–4) were adjusted for baseline context-specific sitting time and change in total physical activity between baseline and follow-up.

## Results

### Sample characteristics

The socio-demographic characteristics, BMI and context-specific sitting at baseline and follow-up are provided in Table 5.

**Table 5:** Sample characteristics at baseline and follow-up.

	BASELINE	FOLLOW-UP
Age (years, mean (SD))	15.0 (1.74)	16.1 (1.73)
Male gender (%)	64.3	64.3
BMI (kg/m^2^, mean (SD))	19.82 (2.96)	20.34 (2.91)
Type of education		
Vocational secondary education (%)	13.9	16.2
Technical secondary education (%)	52.7	55.4
General secondary education (%)	33.3	28.4
TV viewing time (min/average day, median; Q1-Q3)	107.14; 75.36–141.43	90.00; 57.86–107.14
Having parental rules concerning TV time (%)	17.3	11.8
Computer use (min/average day, median; Q1-Q3)	90.00; 45.00–107.14	75.00; 43.57–120.00
Having parental rules concerning computer time (%)	21.9	12.5
Electronic gaming (min/average day, median; Q1-Q3)	57.86; 22.50–107.14	42.86; 7.50–90.00
Having parental rules concerning electronic gaming time (%)	26.1	18.4
Motorized transport (min/average day, median; Q1-Q3)	42.86; 21.43–64.29	42.86; 22.50–72.13

Potential differences between the adolescents who changed class or school (and consequently ‘dropped-out’) and the adolescents who remained in the study were analysed using drop-out analyses. Adolescents who changed class or school showed higher levels of motorized transport (94.63 ± 115.28 vs. 64.80 ± 76.20, p = 006) and consisted of less students following general secondary education (17.8% vs. 33.5%, p = 0.001) compared to adolescents who remained in the study. No differences were found for BMI (p = 0.59), sitting during TV viewing (p = 0.25), sitting during computer use (p = 0.84), sitting during electronic gaming (p = 0.53), self-perceived general health (p = 0.24), depressive symptoms (p = 0.07), parental education (p = 0.20), sex (p = 0.25) and family situation (p = 0.42) between those who changed class or school and those who remained in the study.

### Aim A: Cross-sectional analyses

Table 6 reports the socio-demographic, social-cognitive, physical environmental and health-related cross-sectional correlates of sitting time during TV-viewing, computer use, electronic gaming and motorized transport at baseline.

**Table 6:** Item-specific correlates of sitting during TV time, computer use, electronic gaming and motorized transport (cross-sectional analyses for baseline data).Correlates.

	Dependent variables									
	Sitting during TV viewing		Sitting during computer use		Sitting during motorized transport		Sitting during electronic gaming^a^			
	ExpB^b^ (95% CI)	p	ExpB^b^ (95% CI)	p	ExpB^b^ (95% CI)	p	Logistic model		Gamma model	
							OR of being a person who did sit during gaming (95% CI)	p	ExpB (95% CI)	p
**Socio-demographic variables**										
Family situation	-	-	-	-	-	-	-	-	-	-
Parental education	-	-	-	-	-	-	4.58 (0.56–37.36)	0.16	0.80 (0.60–1.05)	0.11
Sex	-	-	-	-	-	-	0.75 (0.06–8.89)	0.82	1.11 (0.75–1.62)	0.61
Residential area	-	-	-	-	-	-	-	-	-	-
Having siblings	-	-	-	-	-	-	-	-	-	-
Age	-	-	-	-	-	-	0.92 (0.55–1.56)	0.77	1.04 (0.96–1.13)	0.34
Type of education	-	-	-	-	-	-	0.04 (0.003–0.47)	0.01	0.89 (0.66–1.19)	0.42
**Social-cognitive variables**										
Attitude 1	1.16 (1.05–1.28)	0.003	X	X	-	-	X	X	X	X
Attitude 2	-	-	-	-	X	X	1.21 (0.55–2.65)	0.63	1.37 (1.20–1.55)	<0.001
Attitude 3	1.13 (1.05–1.22)	0.001	1.35 (1.21–1.51)	<0.001	-	-	-	-	-	-
Attitude 4	-	-	X	X	X	X	X	X	X	X
Self-efficacy 1	-	-	-	-	-	-	X	X	X	X
Self-efficacy 2	-	-	X	X	X	X	X	X	X	X
Self-efficacy 3	-	-	X	X	X	X	X	X	X	X
Norm	1.18 (1.09–1.27)	<0.001	-	-	1.34 (1.13–1.60)	<0.001	-	-	-	-
Social norm	X	X	X	X	-	-	2.11 (0.77–5.77)	0.14	1.18 (1.05–1.32)	0.004
Social support 1	-	-	-	-	-	-	X	X	X	X
Social support 2	-	-	-	-	-	-	1.67 (0.38–7.34)	0.50	0.87 (0.74–1.02)	0.09
Modelling 1	1.002 (1.001–1.003)	<0.001	-	-	-	-	-	-	-	-
Modelling 2	1.002 (1.001–1.003)	<0.001	1.003 (1.002–1.005)	<0.001	-	-	1.01 (1.00–1.02)	0.24	1.002 (1.00–1.003)	0.02
Modelling 3	X	X	X	X	-	-	X	X	X	X
Modelling 4	X	X	X	X	1.46 (1.02–2.09)	0.04	X	X	X	X
Parental rules	-	-	-	-	X	X	-	-	-	-
**Physical environmental variables**										
TV set	-	-	X	X	X	X	X	X	X	X
Other TV-viewing equipment	-	-	X	X	X	X	X	X	X	X
TV in bedroom	-	-	X	X	X	X	X	X	X	X
Remote controller	1.06 (1.00–1.13)	0.07	X	X	X	X	X	X	X	X
Sitting furniture (couches)	-	-	X	X	X	X	X	X	X	X
PC equipment (desktop & laptop)	X	X	-	-	X	X	X	X	X	X
Other equipment for computer use	X	X	-	-	X	X	X	X	X	X
Equipment for playing games	X	X	X	X	X	X	0.82 (0.57–1.18)	0.28	1.08 (1.02–1.15)	0.006
Other equipment for playing games	X	X	X	X	X	X	-	-	-	-
Motorized vehicles	X	X	X	X	-	-	X	X	X	X
**Health-related variables**										
General health	0.97 (0.89–1.05)	0.46	-	-	-	-	-	-	-	-
Depressive symptoms	1.03 (0.80–1.34)	0.80	-	-	1.43 (0.76–2.69)	0.26	-	-	-	-
BMI	1.04 (0.96–1.12)	0.37	-	-	-	-	-	-	-	-

*Note: Potential correlates were identified by using baseline measurements. A thorough description of the included social-cognitive variables can be found in* Table 1.

^*a*^*number of zero counts = 60.*

^*b*^*expB = the exponent of b can be interpreted as a relative increase (values >1) / decrease (values <1) in sitting time during TV viewing, computer use and motorized transport associated with a one-unit increase in the correlate.*

For sitting during electronic gaming, the interpretation of hurdle models is two-fold. First, a logistic regression was fitted in which the relationship with the probability of a person who did sit during electronic gaming is estimated (Logistic model). Simultaneously, expB can be interpreted as a relative increase/decrease in sitting time during electronic gaming associated with a one-unit increase in the correlate among those who did sit during electronic gaming (Gamma model). “X” indicates correlates not inserted in analyses for context-specific sitting time (i.e. low test-retest reliability, not measured for particular context or not inserted in analysis for particular context). “-” indicates correlates that showed levels of significance p ≥ .10 at the second step. All analyses were adjusted for total physical activity. Abbreviations: OR (odds ratio), PC (computer), TV (television), BMI (body mass index), ns (not significant), CI (confidence interval). p-values of the correlates inserted in the fourth step were reported.

Five social-cognitive variables were significantly associated with *sitting during TV viewing*. A one-unit higher score for ‘I think watching TV is pleasant’ (attitude 1), ‘I enjoy watching TV for many hours at a time’ (attitude 3) and ‘I think that I spend too much time watching TV’ (norm) were associated with respectively 16%, 13% and 18% more sitting per day during TV viewing. Furthermore, 1 minute/day more sitting during TV viewing of the parents/care givers (modelling 1) and siblings (modelling 2) were both associated with 0.2% more sitting per day during TV viewing of the adolescent.

Two social-cognitive variables were significantly associated with *sitting during computer use*. A one-unit higher score for ‘I enjoy using a computer for many hours at a time’ (attitude 3) was associated with 35% more sitting per day during computer use. Furthermore, 1 minute/day more sitting during computer use of the siblings (modelling 2) was associated with 0.3% more sitting per day during computer use of the participant.

Two social-cognitive variables were significantly associated with *sitting during motorized transport*. A one-unit higher score for ‘I think that I spend too much time using motorized transport’ (norm) was associated with 34% more sitting per day during motorized transport. When the most chosen transportation possibility in leisure time from the siblings was motorized transport (modeling 4), 46% more sitting during motorized transport was reported compared to adolescents of whom the siblings preferred active transportation.

One socio-demographic variable in the logistic model and three social-cognitive and one physical environmental variable in the Gamma model were significantly associated with *sitting during electronic gaming*. The logistic model indicated that students from general secondary education had a 96% lower odds of sitting during electronic gaming compared to those attending vocational or technical secondary education. Furthermore, the Gamma model indicated that a one-unit higher score for ‘I enjoy playing computer/video games for many hours at a time’ (attitude 2) and ‘my family members think I spend too much time playing computer/video games’ (social norm) were associated with respectively 37% and 18% minutes/day more sitting during electronic gaming among those who did sit during electronic gaming. Next, 1 minute/day more sitting during electronic gaming of the siblings (modelling 2) was associated with 0.2% minutes/day more sitting during electronic gaming among those who did sit during electronic gaming. Furthermore, a one-unit higher score for the number of ‘equipment for playing games’ was associated with 8% minutes/day more sitting during electronic gaming among those who did sit during electronic gaming.

### Aim B: Longitudinal analyses

For this aim, changes in socio-demographic, social-cognitive, health-related and physical environmental predictors from baseline to follow-up associated with changes from baseline to follow-up in sitting during TV viewing, computer use, electronic gaming and motorized transport were determined (see Table 7).

**Table 7:** Item-specific change scores (predictors) of sitting during TV time, computer use, electronic gaming and motorized transport (longitudinal analyses).

Predictors	Dependent variables							
	Sitting during TV viewing		Sitting during computer use		Sitting during motorized transport		Sitting during electronic gaming	
	B (SE)	p	B (SE)	p	B (SE)	p	B (SE)	p
**Socio-demographic variables**								
Residential area^*α*^								
*Decrease (from BL to FU)*	-4.38 (18.93)	0.82	-	-	-	-	-	-
*Increase (from BL to FU)*	-2.89 (18.45)	0.88	-	-	-	-	-	-
Type of education^β^								
*Change to general secondary education (from BL to FU)*	-	-	-	-	-	-	-	-
*Change to vocational or technical secondary education (from BL to FU)*	-	-	-	-	-	-	-	-
**Social-cognitive variables**								
Attitude 1	14.39 (4.36)	<0.001	16.76 (6.14)	0.006	-	-	-	-
Attitude 2	-	-	-	-	X	X	-	-
Attitude 3	-	-	X	X	-	-	-	-
Attitude 4	9.69 (3.78)	0.02	-	-	X	X	-	-
Self-efficacy 1	-	-	X	X	-	-	X	X
Self-efficacy 2	-	-	-	-	X	X	X	X
Self-efficacy 3	-	-	X	X	X	X	X	X
Norm	-	-	-	-	-	-	-	-
Social norm	-	-	X	X	-	-	-	-
Social support 1	-	-	10.64 (5.77)	0.07	-	-	-	-
Social support 2	-	-	-4.67 (6.96)	0.50	-	-	-	-
Modelling 1	0.19 (0.06)	0.002	-	-	-	-	-	-
Modelling 2	0.19 (0.05)	<0.001	0.29 (0.06)	<0.001	11.89 (10.43)	0.25	-	-
Modelling 3	X	X	X	X	12.56 (11.74)	0.28	X	X
Modelling 4	X	X	X	X	-	-	X	X
Parental rules	-7.03 (11.96)	0.56	-	-	X	X	-22.31 (16.31)	0.17
**Physical environmental variables**								
TV set	-	-	X	X	X	X	X	X
Other TV-viewing equipment	-	-	X	X	X	X	X	X
TV in bedroom	-	-	X	X	X	X	X	X
Remote controller	-	-	X	X	X	X	X	X
Sitting furniture (couches)	-	-	X	X	X	X	X	X
PC equipment (desktop & laptop)	X	X	-	-	X	X	X	X
Other equipment for computer use	X	X	-	-	X	X	X	X
Equipment for playing games	X	X	X	X	X	X	-	-
Other equipment for playing games	X	X	X	X	X	X	-	-
Motorized vehicles	X	X	X	X	-	-	X	X
**Health-related variables**								
General health	-	-	-	-	-	-	-	-
Depressive symptoms								
*Developing depressive symptoms (from BL to FU)*	-	-	-	-	-	-	-	-
*Disappearance of depressive symptoms (from BL to FU)*	-	-	-	-	-	-	-	-
BMI	-	-	-	-	-	-	7.84 (8.75)	0.37

*Note: A thorough description of the included social-cognitive variables can be found in* Table 1. *“X” indicates predictors not inserted in the analyses for context-specific sitting time (i.e. low test-retest reliability, not measured for particular context or not inserted in analysis for particular context). “-” indicates predictors that showed levels of significance p ≥ .10 at the second step. All analyses were adjusted for baseline context-specific sitting time and change score for total physical activity. Abbreviations: BL (baseline), FU (follow-up), PC (computer), TV (television), SE (standard error), BMI (body mass index), ns (not significant).p-values of the predictors inserted in the fourth step were reported. B-values can be interpreted as change in minutes/day of context-specific sitting time, in which positive values indicate an increase in context-specific sitting time and negative values indicate a decrease in context-specific sitting time (expressed in minutes/day). The reference category for residential area*^*α*^ *and type of education*^*β*^ *was ‘being in the stable group’.*

Four social-cognitive change scores were significantly associated with *change in sitting during TV viewing*. An increase from baseline to follow-up with one unit on the five-point Likert scale for ‘I think watching TV is pleasant’ (attitude 1) and ‘watching TV is my way to relax’ (attitude 4) were associated with respectively 14.4 minutes/day and 9.7 minutes/day more sitting during TV viewing at follow-up. Furthermore, an increase from baseline to follow-up with 1 minute/day sitting during TV viewing of the parents/care givers (modelling 1) and siblings (modelling 2) were both associated with 0.2 minutes/day more sitting during TV viewing at follow-up of the participant.

Two social-cognitive change scores were significantly associated with *change in sitting during computer use*. An increase from baseline to follow-up with one unit on the five-point Likert scale for ‘I think using a computer is pleasant in leisure time’ (attitude 1) was associated with 16.8 minutes/day more sitting during computer use at follow-up. Also, an increase from baseline to follow-up with 1 minute/day sitting during computer use of the siblings (modelling 2) was associated with 0.3 minutes/day more sitting during computer use at follow-up of the participant.

No significant associations were found between changes in ecological variables and *changes in sitting during motorized transport* and during *electronic gaming*.

## Discussion

This study examined cross-sectional and longitudinal associations between ecological variables and sitting during TV viewing, computer use, motorized transport and electronic gaming among Belgian adolescents. The results increase the knowledge on sitting time in adolescents, as to date limited evidence is available on multilevel variables (i.e. socio-demographic, social-cognitive, physical environmental and health-related variables) associated with context-specific leisure sitting time [23].

In the cross-sectional and longitudinal analyses different associated variables were identified for sitting during TV viewing, computer use, motorized transport and electronic gaming. Previously, Babey et al. [20] concluded that different cross-sectional correlates were found for TV viewing and computer use. However, the present findings revealed that three variables were found to be associated with several of the included contexts of sitting in the cross-sectional analyses; modelling of siblings during TV viewing, computer use, motorized transport and electronic gaming; norm for TV viewing and motorized transport; and attitude ‘enjoyment’ for TV viewing and computer use. In the longitudinal analyses, two out of the four significant predictors for changes in TV viewing were also associated with changes in computer use, i.e. attitude ‘pleasant’ and modelling siblings. These consistent correlates/predictors of sitting time in different contexts may be important for developing future interventions, as targeting one variable could change behaviours in multiple contexts. Subsequently, only focusing on the individual in future interventions may not be effective to change adolescents’ sitting time, highlighting the importance of a socio-ecological approach [22].

Further, social-cognitive variables were most frequently associated with the included context-specific sitting outcomes compared with the included socio-demographic, physical environmental and health-related variables, especially for the screen-related contexts (TV viewing, computer use and electronic gaming). Importantly, for the longitudinal analyses, only changes in variables at the social-cognitive level were associated with changes in sitting time during TV viewing and computer use. In line with the present limited findings of variables belonging to physical environmental level, a systematic review of Stierlin et al. [23] concluded that no or inconsistent evidence was available for associations between environmental variables and different measures of sitting. Present cross-sectional and longitudinal results showed the importance of modelling of relatives concerning context-specific sitting. The results highlighted the importance of the siblings’ behaviour for sitting during screen-related behaviours. In line with these findings, Granich et al. [37] concluded in their qualitative study that siblings, especially those of similar sex, had an important influence on TV viewing, electronic games and computer use among 11-to-12-year-olds. In the present study, an increase of 60 minutes in sitting during TV viewing or computer use of the siblings over a one-year follow-up period was associated with 12 minutes more sitting during TV viewing and 18 minutes more sitting during computer use at follow-up of the adolescent. Consequently, siblings may play an important role when aiming to minimize context-specific sitting in interventions among adolescents, especially screen-related behaviours, which was also stressed in previous qualitative research [37]. Also, the present strong association between adolescents’ and their parents’ time spent sitting during TV viewing might indicate that TV viewing is a family habit. The present longitudinal findings highlighted that an increase of 60 minutes in sitting during TV viewing of the parents/care givers from baseline to follow-up was associated with 12 minutes more sitting during TV viewing at follow-up of the adolescent. Previous evidence already showed that parents might act as a role model, and therefore, parents should be aware of their impact on the TV viewing behaviour of their children [37]. Importantly, Tandon et al. [38] showed that children spent more time sitting during TV viewing together with their parents or siblings compared to engagement in physical activity with their relatives. These and the current findings highlight the need of family-based interventions focusing on changing screen-related sitting time.

The present findings also showed that attitude is a key construct of screen-related sitting. The cross-sectional results highlighted that perceived enjoyment in screen-related sitting was positively associated with sitting time during these activities. These findings support a qualitative study conducted in the UK that concluded that future intervention developers should acknowledge the perceived enjoyment of screen-related sitting among 10-to-11-year-olds [39]. Furthermore, for TV viewing and computer use, an increase from baseline to follow-up in finding these behaviours pleasant predicted more sitting during TV viewing and computer use at follow-up. For TV viewing, perceiving TV watching as a way to relax was found to be significantly associated in the longitudinal analyses. As sitting during TV viewing and computer use were associated with a strong positive attitude towards these contexts of sitting, it can be hard to replace these behaviours with physical activity in future interventions. Future studies should identify why adolescents have strong positive attitude, as for example the positive attitude towards sitting during computer use may be more explained by the use of new digital media (tablets and smartphone) and less by traditional computer use on laptop or desktop. Also, social norm was positively associated with sitting during electronic gaming in the cross-sectional analyses. In line with this finding, a Belgian study found that the perception of family social norm concerning internet use was positively associated with higher levels of internet use during leisure time among adults [26]. Interestingly in the present study, parental rules concerning time adolescents spent sitting during TV viewing, computer use and electronic gaming were however not associated with context-specific sitting time. Cillero et al. [40] found that for secondary schoolchildren, co-viewing practices with family members were important regarding TV viewing, computer playing and console playing while parental rules concerning screen-related sitting may be more relevant for primary school-aged children. According to the latter study, screen-related sitting time of adolescents might to some extent be explained by less parental rules concerning screen-related sitting and more co-viewing practices in the family [40], a reasoning supported by the current findings. In summary, the results showed that modelling of parents/care givers and/or siblings and attitude (pleasant and/or relaxing) showed to be both significantly associated with sitting during TV viewing and computer use in the cross-sectional and longitudinal analyses, highlighting the importance of these variables for future interventions. Different strategies, based on the present findings, may be suitable to minimize sitting during TV viewing and computer use. These include minimizing parental screen time and minimizing screen time of siblings. Furthermore, including activity breaks during TV viewing and computer use may be advised regarding the strong positive attitude towards these behaviours. These suggestions were also addressed in a recent qualitative meta-synthesis, as until now there is only limited evidence of existing interventions that successfully changed screen time of youth [41].

A first limitation of the present study is the lower test-retest reliability for sitting during motorized transport and electronic gaming. However, this might be explained by the between-week variability of these behaviours, as the test and retest did not record the same period [12]. Secondly, there was a substantial drop-out rate (26.3%) due to practical reasons. Adolescents who changed class or school (and were therefore not included at follow-up) differed in certain characteristics (motorized transport and type of education) compared to the adolescents who remained in the study. Furthermore, future research should include schools spread across different regions in Flanders. Thirdly, (total) sitting time was not measured objectively which may have resulted in recall bias or social desirable answers. Fourthly, the majority of the present study sample was male and followed technical secondary education. Thus, additional research is necessary to verify the generalizability of the current findings. Finally, the present study revealed that the cross-sectional correlates of context-specific sitting mostly differed from the longitudinal predictors of these contexts. This may be explained by the limited variance over time in the reported answers of the included variables. Therefore, additional experimental research is recommended to identify if changing cross-sectional correlates also lead to changes in context-specific sitting time (e.g. intervention studies). Still, the longitudinal predictors are more likely to introduce changes in sitting time compared with the cross-sectional correlates, as changes in these variables already predicted changes in sitting time.

An important strength of the present study is the inclusion of a range of modifiable and multilevel potential associated variables of context-specific sitting time among adolescents. Furthermore, this is one of the first studies which included both cross-sectional and longitudinal analyses, so that comparisons between cross-sectional correlates and longitudinal predictors were possible. Lastly, the inclusion of item-specific social-cognitive variables was of added value, as different social-cognitive variables belonging to one construct (e.g. attitude 1–4) measure slightly different, but all important aspects of the associated construct (e.g. attitude). Consequently, item-specific variable information was relevant for the interpretation concerning the set-up of more effective interventions in the future.

## Conclusions

The present study found different correlates and/or predictors for the various contexts of sitting time among adolescents. For some contexts, similar associated variables were however identified, especially for TV viewing and computer use. These consistent findings for different contexts may provide evidence for intervention developers to have a potential impact in multiple contexts of sitting. In general, correlates and predictors at the social-cognitive level were most frequently related to context-specific sitting. Limited evidence was found at the socio-demographic and physical environmental level in both the cross-sectional and longitudinal analyses. No associations were found at the health-related level. Further, intervention developers should be aware that variables which were cross-sectionally associated with context-specific sitting time may not all cause change in context-specific sitting time. The longitudinal analyses solely showed predictors identified for sitting during TV viewing and computer use. Intervention developers should acknowledge the following issues, based on the results of the longitudinal analyses, while setting up future interventions to minimize context-specific sitting time. First, experiencing more pleasure in TV viewing and computer use and more sitting time of siblings during TV viewing and computer use will lead to more sitting during these contexts. Next, TV viewing of the parents or care givers should also be targeted in order to limit TV viewing behaviour of the adolescents. Lastly, alternative habits to relax should be identified to reduce TV time. Consequently, future interventions should include both an intrapersonal and interpersonal component, as context-specific sitting may be hard to minimize while only focussing on the individual. More longitudinal research is necessary to confirm the findings of the present study. Randomized controlled experimental study designs should be implemented in future studies in order to examine whether changing the significant longitudinal predictors of the present study actually lead to changes in sitting time.

## References

[1] Barnes J, Behrens TK, Benden ME, Biddle S, Bond D, Brassard P et al Letter to the Editor: Standardized use of the terms "sedentary" and "sedentary behaviours". Appl Physiol Nutr Me. 2012;37(3):540–542. 10.1139/h2012-024 22540258

[2] Owen N, Salmon J, Koohsari MJ, Turrell G, Giles-Corti B. Sedentary behaviour and health: mapping environmental and social contexts to underpin chronic disease prevention. Brit J Sport Med. 2014;48(3):174–7. 10.1136/bjsports-2013-093107 24415410

[3] de Rezende LFM, Lopes MR, Rey-Lopez JP, Matsudo VKR, Luiz OD. Sedentary Behavior and Health Outcomes: An Overview of Systematic Reviews. Plos One. 2014;9(8):e105620 10.1371/journal.pone.0105620 25144686 PMC4140795

[4] Ivuskans A, Maestu J, Jurimae T, Latt E, Purge P, Saar M et al Sedentary time has a negative influence on bone mineral parameters in peripubertal boys: a 1-year prospective study. J. Bone Miner. Metab. 2015;33(1):85–92. 10.1007/s00774-013-0556-4 24549738

[5] Grontved A, Singhammer J, Froberg K, Moller NC, Pan A, Pfeiffer KA et al A prospective study of screen time in adolescence and depression symptoms in young adulthood. Prev Med. 2015;81:108–13. 10.1016/j.ypmed.2015.08.009 26303369

[6] Wennberg P, Gustafsson PE, Howard B, Wennberg M, Hammarstrom A. Television viewing over the life course and the metabolic syndrome in mid-adulthood: a longitudinal population-based study. J Epidemiol Community Health. 2014;68(10):928–33. 10.1136/jech-2013-203504 24663095

[7] Lee PH. Association between adolescents' physical activity and sedentary behaviors with change in BMI and risk of type 2 diabetes. Plos One. 2014;9(10):e110732 10.1371/journal.pone.0110732 25340773 PMC4207744

[8] Salmon J. Novel Strategies to Promote Children's Physical Activities and Reduce Sedentary Behavior. J Phys Act Health. 2010;7:S299–S306. 21116014 10.1123/jpah.7.s3.s299

[9] Saunders TJ, Tremblay MS, Mathieu ME, Henderson M, O'Loughlin J, Tremblay A et al Associations of sedentary behavior, sedentary bouts and breaks in sedentary time with cardiometabolic risk in children with a family history of obesity. Plos One. 2013;8(11):e79143 10.1371/journal.pone.0079143 24278117 PMC3835898

[10] Dowd KP, Harrington DM, Hannigan A, Donnelly AE. Light-intensity physical activity is associated with adiposity in adolescent females. Med. Sci. Sports Exerc. 2014;46(12):2295–300. 10.1249/MSS.0000000000000357 24797308

[11] McManus AM, Ainslie PN, Green DJ, Simair RG, Smith K, Lewis N. Impact of prolonged sitting on vascular function in young girls. Exp Physiol. 2015;100(11):1379–87. 10.1113/EP085355 26370881

[12] Busschaert C, De Bourdeaudhuij I, Van Holle V, Chastin SF, Cardon G, De Cocker K. Reliability and validity of three questionnaires measuring context-specific sedentary behaviour and associated correlates in adolescents, adults and older adults. Int. J. Behav. Nutr. Phys. Act. 2015;12:117 10.1186/s12966-015-0277-2 26381488 PMC4574538

[13] Rideout VJ, Foehr UG, Roberts DF. GenerationM[superscript 2]:Media in the Lives of 8-to 18-Year-Olds. Henry J Kaiser FamFound. 2010.

[14] Babic MJ, Morgan PJ, Plotnikoff RC, Lonsdale C, Eather N, Skinner G et al Rationale and study protocol for 'Switch-off 4 Healthy Minds' (S4HM): a cluster randomized controlled trial to reduce recreational screen time in adolescents. Contemp Clin Trials. 2015;40:150–8. 10.1016/j.cct.2014.12.001 25500220

[15] Grize L, Bringolf-Isler B, Martin E, Braun-Fahrlander C. Trend in active transportation to school among Swiss school children and its associated factors: three cross-sectional surveys 1994, 2000 and 2005. Int J Behav Nutr Phy. 2010;7:28. 10.1186/1479-5868-7-28 PMC2867987 20398320

[16] Chastin SFM, Schwarz U, Skelton DA. Development of a Consensus Taxonomy of Sedentary Behaviors (SIT): Report of Delphi Round 1. Plos One. 2013;8(12):e82313 10.1371/journal.pone.0082313 24312653 PMC3847079

[17] Saunders TJ, Chaput JP, Tremblay MS. Sedentary Behaviour as an Emerging Risk Factor for Cardiometabolic Diseases in Children and Youth. Can J Diabetes. 2014;38(1):53–61. 10.1016/j.jcjd.2013.08.266 24485214

[18] Esteban-Cornejo I, Martinez-Gomez D, Sallis JF, Cabanas-Sanchez V, Fernandez-Santos J, Castro-Pinero J et al Objectively measured and self-reported leisure-time sedentary behavior and academic performance in youth: The UP&DOWN Study. Prev Med. 2015;77:106–11. 10.1016/j.ypmed.2015.05.013 26013994

[19] Hamar P, Biddle S, Soos I, Takacs B, Huszar A. The prevalence of sedentary behaviours and physical activity in Hungarian youth. Eur J Public Health. 2010;20(1):85–90. 10.1093/eurpub/ckp100 19587226

[20] Babey SH, Hastert TA, Wolstein J. Adolescent Sedentary Behaviors: Correlates Differ for Television Viewing and Computer Use. J Adolescent Health. 2013;52(1):70–6. 10.1016/j.jadohealth.2012.05.001 PMC3786734 23260837

[21] Busschaert C, Cardon G, Van Cauwenberg J, Maes L, Van Damme J, Hublet A et al Tracking and Predictors of Screen Time From Early Adolescence to Early Adulthood: A 10-Year Follow-up Study. J. Adolesc. Health. 2015;56:440–448. 10.1016/j.jadohealth.2014.11.016 25636817

[22] Owen N, Sugiyama T, Eakin EE, Gardiner PA, Tremblay MS, Sallis JF. Adults' Sedentary Behavior Determinants and Interventions. Am J Prev Med. 2011;41(2):189–96. 10.1016/j.amepre.2011.05.013 21767727

[23] Stierlin AS, De Lepeleere S, Cardon G, Dargent-Molina P, Hoffmann B, Murphy MH et al A systematic review of determinants of sedentary behaviour in youth: a DEDIPAC-study. Int. J. Behav. Nutr. Phys. Act. 2015;12:133 10.1186/s12966-015-0291-4 26453175 PMC4600309

[24] Ridgers ND, Timperio A, Crawford D, Salmon J. What factors are associated with adolescents' school break time physical activity and sedentary time? Plos One. 2013;8(2):e56838 10.1371/journal.pone.0056838 23418606 PMC3572081

[25] Simons D, Clarys P, De Bourdeaudhuij I, de Geus B, Vandelanotte C, Deforche B. Factors influencing mode of transport in older adolescents: a qualitative study. Bmc Public Health. 2013;13:323 10.1186/1471-2458-13-323 23574974 PMC3626670

[26] Van Dyck D, Cardon G, Deforche B, Owen N, De Cocker K, Wijndaele K et al Socio-demographic, psychosocial and home-environmental attributes associated with adults' domestic screen time. Bmc Public Health. 2011;11:668 10.1186/1471-2458-11-668 21864412 PMC3175191

[27] Richardson LP, Rockhill C, Russo JE, Grossman DC, Richards J, McCarty C et al Evaluation of the PHQ-2 as a Brief Screen for Detecting Major Depression Among Adolescents. Pediatrics. 2010;125(5):E1097–E103. 10.1542/peds.2009-2712 20368315 PMC3100798

[28] Borner I, Braunstein JW, Victor RS, Pollack J. Evaluation of a 2-Question Screening Tool for Detecting Depression in Adolescents in Primary Care. Clin Pediatr. 2010;49(10):947–53. 10.1177/0009922810370203 20724330

[29] Philippaerts RM, Matton L, Wijndaele K, Balduck AL, De Bourdeaudhuij I, Lefevre J. Validity of a physical activity computer questionnaire in 12-to 18-year-old boys and girls. Int J Sports Med. 2006;27(2):131–6. 10.1055/s-2005-837619 16475059

[30] Roelants M, Hauspie R: Groeicurven 2–20 jaar, Vlaanderen 2004, [Growth charts 2–20 years, Flanders 2004] 2004 [http://www.vub.ac.be/groeicurven]. Laboratorium voor Antropogenetica, Vrije Universiteit Brussel, Brussel

[31] Cole TJ: The LMS method for constructing normalized growth standards. Eur J Clin Nutr 1990, 44:45–60. 2354692

[32] Cole TJ, Green PJ: Smoothing reference centile curves: the LMS method and penalized likelihood. Stat Med 1992, 11:1305–1319. 1518992 10.1002/sim.4780111005

[33] Plewis I, Fielding A. What is multi-level modelling for? A critical response to Gorard (2003). Brit J Educ Stud. 2003;51(4):408–19.

[34] Ghasemi A, Zahediasl S. Normality Tests for Statistical Analysis: A Guide for Non-Statisticians. Int J Endocrinol Metab. 2012;10(2):486–489. 10.5812/ijem.3505 23843808 PMC3693611

[35] De Bourdeaudhuij I, Lefevre J, Deforche B, Wijndaele K, Matton L, Philippaerts R. Physical activity and psychosocial correlates in normal weight and overweight 11 to 19 year olds. Obes Res. 2005;13(6):1097–105. 10.1038/oby.2005.128 15976153

[36] De Bourdeaudhuij I, Sallis J. Relative contribution of psychosocial variables to the explanation of physical activity in three population-based adult samples. Prev Med. 2002;34(2):279–88. 10.1006/pmed.2001.0979 11817925

[37] Granich J, Rosenberg M, Knuiman M, Timperio A. Understanding children's sedentary behaviour: a qualitative study of the family home environment. Health Educ Res. 2010;25(2):199–210. 10.1093/her/cyn025 18502732

[38] Tandon PS, Zhou C, Sallis JF, Cain KL, Frank LD, Saelens BE. Home environment relationships with children's physical activity, sedentary time, and screen time by socioeconomic status. Int. J. Behav. Nutr. Phys. Act. 2012;9:88 10.1186/1479-5868-9-88 22835155 PMC3413573

[39] Sebire SJ, Jago R, Gorely T, Cillero IH, Biddle SJH. "If there wasn't the technology then I would probably be out everyday": A qualitative study of children's strategies to reduce their screen viewing. Prev Med. 2011;53(4–5):303–8. 10.1016/j.ypmed.2011.08.019 21884723

[40] Cillero IH, Jago R, Sebire S. Individual and social predictors of screen-viewing among Spanish school children. Eur J Pediatr. 2011;170(1):93–102. 10.1007/s00431-010-1276-6 20814697

[41] Minges KE, Owen N, Salmon J, Chao A, Dunstan DW, Whittemore R. Reducing youth screen time: qualitative metasynthesis of findings on barriers and facilitators. Health Psychol. 2015;34(4):381–97. 10.1037/hea0000172 25822054 PMC4456186

